# MET expression and copy number status in clear-cell renal cell carcinoma: prognostic value and potential predictive marker

**DOI:** 10.18632/oncotarget.13540

**Published:** 2016-11-24

**Authors:** Stephan Macher-Goeppinger, Martina Keith, Volker Endris, Roland Penzel, Katrin E. Tagscherer, Sascha Pahernik, Markus Hohenfellner, Humphrey Gardner, Carsten Grüllich, Peter Schirmacher, Wilfried Roth

**Affiliations:** ^1^ Institute of Pathology, University Hospital Heidelberg, Heidelberg, Germany; ^2^ Molecular Tumor Pathology, German Cancer Research Center (DKFZ), Heidelberg, Germany; ^3^ Institute of Pathology, University Medical Center Mainz, Mainz, Germany; ^4^ Department of Urology, University Hospital Heidelberg, Heidelberg, Germany; ^5^ Translational Medicine, Early Clinical Development, AstraZeneca, Gatehouse Park, Waltham, MA, USA; ^6^ Department of Medical Oncology, National Center for Tumor Diseases Heidelberg, University Hospital Heidelberg, Heidelberg, Germany

**Keywords:** renal cell carcinoma, MET, HGF, targeted therapy, biomarker

## Abstract

Multiple targeted therapy for advanced clear-cell renal cell carcinoma (RCC) has substantially improved patient outcome, but complete remission is uncommon and many tumors eventually develop resistance. Mechanistic, preclinical, and early clinical data highlight c-Met / hepatocyte growth factor receptor as a promising target for RCC therapeutic agents.

We have examined MET expression, frequency of *MET* gene copy gains and *MET* gene mutation in a large, hospital-based series of renal cell carcinomas with long-term follow-up information.

Out of a total of 572 clear-cell RCC, only 17% were negative for MET expression whereas 32% showed high protein levels. High MET expression and *MET* copy number gains were associated with an aggressive phenotype and an unfavorable patient outcome. Elevated protein levels in absence of gene amplification were not attributed to mutations, based on results of targeted next-generation sequencing.

Our data reveal that clear-cell RCC with MET upregulation show an aggressive behavior and *MET* copy number increase is evident in a substantial percentage of patients with high-grade carcinomas and metastatic disease. Diagnostic assessment of MET expression and amplification may be of predictive value to guide targeted therapy against MET signaling in patients with clear-cell RCC.

## INTRODUCTION

Treatment of metastatic renal cell carcinoma (mRCC) has dramatically changed over the last decade and multiple targeted therapies have replaced IL-2 and IFN-α immunotherapy as the primary treatment option. These new agents, such as Sorafenib, Sunitinib or Temsirolimus, mainly target two pathways, vascular endothelial growth factor (VEGF) signaling and the mammalian target of rapamycin (mTOR) [1, 2, 3, 4, 5, 6]. Although targeted agents have substantially improved patient outcomes, 14.080 deaths due to kidney and renal pelvic cancer are estimated for 2015 in the United States [7], not least because complete response is rare and many tumors eventually develop resistance [8, 9, 10]. Therefore, novel treatment approaches are urgently needed and efforts to identify novel therapeutic targets based on molecular tumor characteristics are necessary.

*MET* encodes for a receptor tyrosine kinase (RTK) called c-Met or hepatocyte growth factor receptor (HGFR). Cells of epithelial origin widely present HGFR on their surface and overexpression of HGFR is common in carcinomas [11]. The ligand for HGFR is hepatocyte growth factor/scatter factor (HGF/SF) and binding induces recruitment of several signaling effectors. Downstream signaling of HGFR includes activation of MAPK and PI3K-AKT pathway and induction of the vascular endothelial growth factor (VEGF) and in consequence evokes a variety of pleiotropic pro-tumorigenic responses, like cell migration, proliferation and angiogenesis [12].

Mechanisms of *MET* activation include mutation and amplification. Activating germline *MET* mutations have been observed in patients with hereditary papillary RCCs (papRCC) and in 13% of sporadic papillary RCC [13]. Moreover, somatic mutations of *MET* have been reported in head and neck squamous cell carcinomas [14], lung adenocarcinomas [15], and childhood hepatocellular carcinomas [16]. *MET* amplifications have been reported in colorectal, gastric, esophageal, lung and clear-cell ovarian cancer [17, 18, 19, 20, 21]. High HGFR protein expression is frequently observed in carcinomas with aggressive phenotype and associated with poor prognosis in non-small cell lung, ovarian and colorectal cancer [22, 23, 24]. Besides papillary RCC, MET upregulation has also been observed in clear-cell RCC (ccRCC) and association with poor pathologic features and unfavorable prognosis has been described [25, 26]. However, larger studies addressing HGFR protein levels and genetic alterations are missing.

Therefore, we systematically assessed the molecular status of MET in ccRCC in correlation with clinical features in a large, hospital-based series with long-term follow-up information and show that elevated HGFR expression and MET amplifications are evident in a substantial percentage of metastatic and/or aggressive ccRCCs and emerge de novo in RCC metastasis.

## RESULTS

### Immunohistochemistry and CISH Analyses

**Figure 1:**
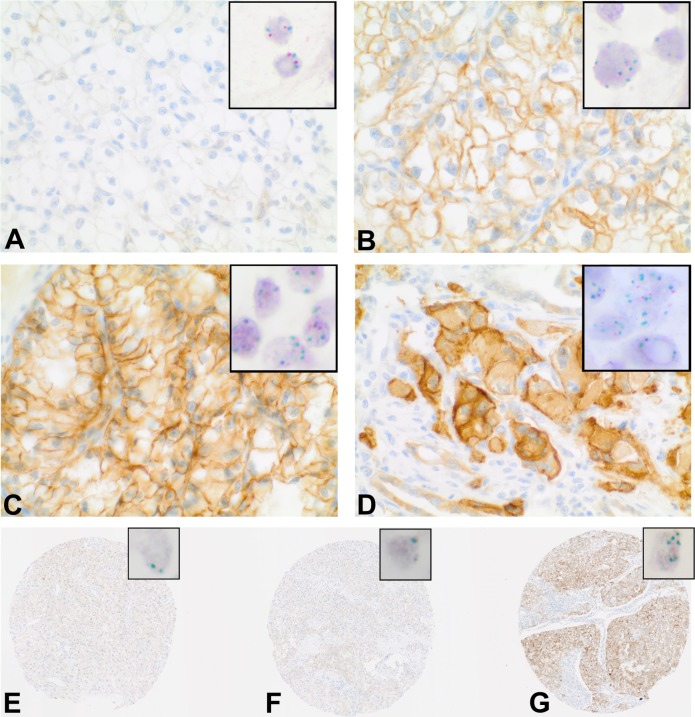
HGFR expression and *MET* copy number in clear-cell RCC **A-G.** Immunohistochemical demonstration of HGFR expression; Insert: corresponding Chromogenic-*in-situ*-Hybridisation (CISH) with centromeric region of chromosome 7 (red) and *MET* signals (green) in the nuclei. **A-D.** HGFR expression and *MET* copy numbers in primary ccRCC. (A) No elevated protein levels, no copy number increase. (B) Immunoreactive score (IRS) = 8, *MET* copy number >4. (C) IRS = 12; *MET* copy number >4. (D) IRS = 12, *MET* copy number >8. E-G: MET status in different regions of the primary tumor and matching metastasis. **E+F.** Primary tumor, no elevated protein levels and no copy number increase. (G) Metastasis, IRS = 12, *MET* copy number >4.

Immunostains and Chromogenic-*in-situ*-Hybridisation (CISH) were performed on tissue microarrays (TMA) containing tumor and corresponding normal renal tissue from 932 patients with renal cell carcinomas. The study is focused on 763 clear-cell RCC. In total 572 cases could be successfully scored for both, HGFR expression by immunohistochemistry and *MET* copy number by CISH analysis. The remaining cases were excluded from further analyses either because of insufficient tumor tissue, poor tissue preservation or missing patient information. Figure 1(A-D) depicts immunohistochemical HGFR expression and corresponding *MET* CISH results. The clinical and pathological features of the study population are summarized in Table 1.

**Table 1:** Clinicopathological characteristics of the study population

Variable	n(%)
**Study Population**	572
**Fuhrman Grade**	
1	163(29)
2	317(55)
3	92(16)
4	2(0)
**Primary tumor**	
pT1	313(55)
pT2	46(8)
pT3	191(33)
pT4	22(4)
**Synchronous distant metastasis**	
yes	91(15)
no	481(84)
**Local lymphnode metastasis**	
pN1	35(6)
pN0	331(58)
pNx	206(36)
**Sex**	
female	234(41)
male	338(59)
**Age at surgery**	
>65	251(44)
≤65	321(56)
**ECOG**	
0	353(62)
≥1	219(38)

HGFR was immunohistochemically detected in a total of 83% cases (476/572), 51% (292/572) showed low (defined as immunoreactive score (IRS) < 6) and 32% (184/572) high cytoplasmic positivity (defined as IRS ≥ 6), the remaining 96 cases were completely negative (17%). *MET* copy number gains could be detected in 310 cases, 47% (272/572) tumors exhibited >2-4 *MET* copies per nucleus and another 7% (38/572) tumors more than 4 copies per nucleus, the remaining 46% (262/572) cases showed 2 copies per nucleus. Increase of MET/centromer-7 ratio > 2 was only seen in 7 tumors, all being positive by immunohistochemistry; furthermore 37 out of 38 tumors with more than 4 copies per nucleus were positive by HGFR immunohistochemistry (Supplementary Figure S1A+B). In turn, out of 187 tumors with high HGFR protein levels only 5 tumors showed *MET*/centromer-7 ratio >2, respectively 141 tumors an average *MET* copy number >2, the remaining tumors (n=46) 2 *MET* copies/nucleus. However, the protein levels of HGFR and *MET* copy number showed a weak positive correlation (Spearman's rank correlation rho=0.377; p < 0.001, Supplementary Figure S1C).

### HGFR expression / MET copy numbers and tumor progression

As MET signaling has been reported to be pivotal in development of cancer and metastasis [12] we created another TMA including tissue of 18 primary ccRCCs and corresponding metastasis. Out of 15 primary ccRCCs with low/no HGFR expression, high HGFR protein levels were detected in 6 corresponding metastases. Figure 1(E-G) demonstrates strong increase of HGFR expression in the metastasis of a primarily HGFR-negative ccRCC. CISH analyses revealed that increase of protein levels was accompanied by *MET* copy number gains in 5 out of these 6 cases with an average of two *MET* copies per nucleus in the primary tumor and an average of 4.4 *MET* copies per nucleus in the metastasis. The remaining case showed stable *MET* copy numbers in primary tumor and metastasis.

### Mutation analysis

As increased HGFR protein levels were observed in the absence of *MET* copy number gains, HGFR overexpression has to be based on other mechanisms than amplification. As mutations that inactivate the Cbl binding site lead to constitutive HGFR expression [27], we checked for *MET* mutations by targeted next-generation sequencing in 9 tumors with high HGFR protein levels and 2 *MET* copies/nucleus and 2 tumors with high HGFR protein levels and gains of *MET* copy numbers. No *MET* mutations were found using Ion AmpliSeqTM Cancer Hotspot Panel v2 comprising exon 2, 11, 14, 16, and 19 of the MET gene. Besides *VHL* mutations, we did not identify other recurrent mutations in the examined genes.

### Comparison of HGFR expression / MET copy numbers with Clinical and Pathological Features

**Figure 2:**
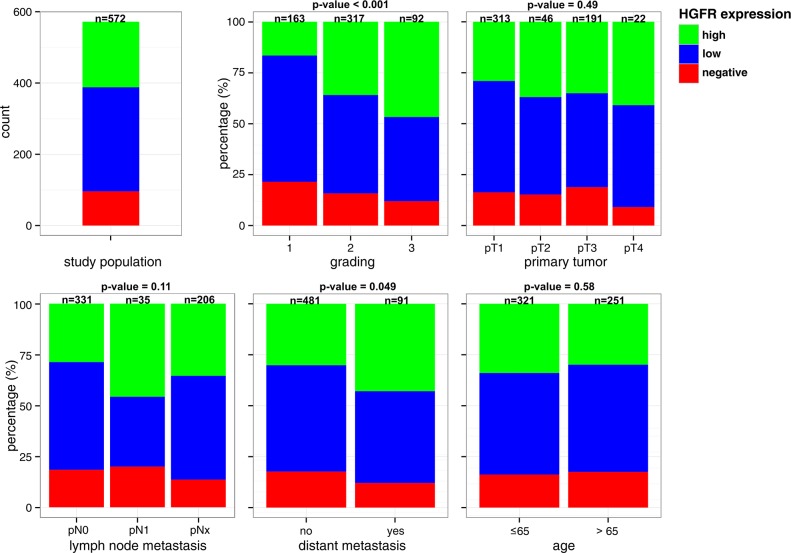
Comparison of HGFR expression with Clinical and Pathological Features

The proportion of tumors positive for HGFR by immunohistochemistry increased with dedifferentiation (P < 0.001) and occurrence of distant metastasis (P = 0.049) (Figure 2). For example, percentage of tumors with high HGFR expression was 17% in G1 compared to 46% in G3 carcinomas. No consistent association of HGFR expression and tumor extent, lymph node metastasis and patient age was observed.

**Figure 3:**
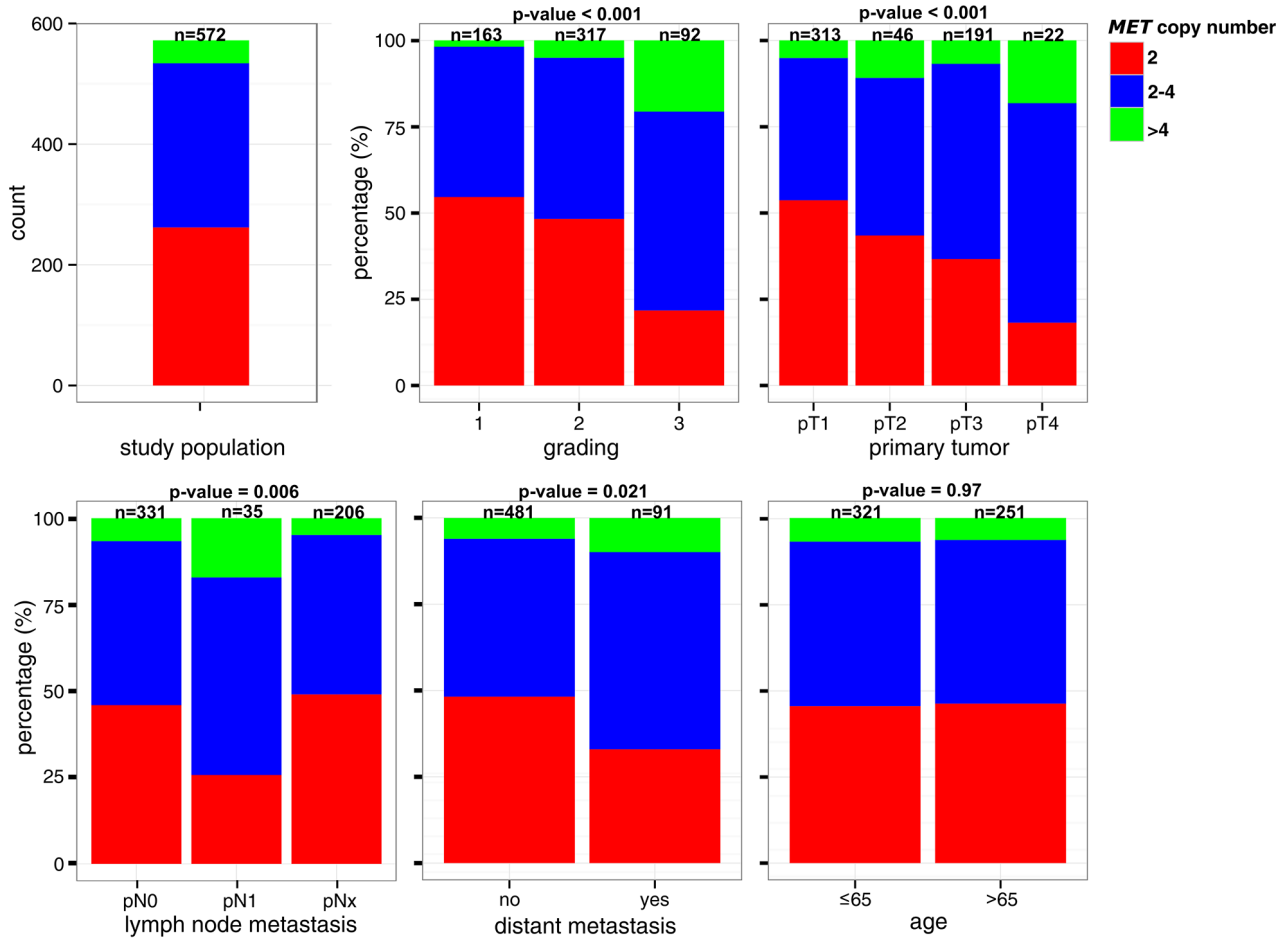
Comparison of *MET* copy number status with Clinical and Pathological Features

*MET* copy number gains were accompanied by dedifferentiation (P < 0.001), higher tumor extent (P < 0.001), positive lymphnode status (P = 0.006), and occurrence of distant metastasis (P = 0.02) (Figure 3). For instance, the proportion of tumors with more than four *MET* copies/nucleus increased about 10 times in high-grade carcinomas compared to low-grade carcinomas (20% vs 2%). Whereas more than half of T1-carcinomas (54%) showed two *MET* copy numbers/nucleus, tumors without copy number gains represent only 18% of locally advanced (T4) carcinomas. No consistent association between *MET* copy numbers and patient age was observed.

### HGFR expression / MET copy numbers and patient prognosis

The median time of follow-up was 8 years (mean 7.8, min 0.014, max 23.7 years), among the 572 patients, 189 had died from ccRCC by the end of the follow-up.

**Figure 4:**
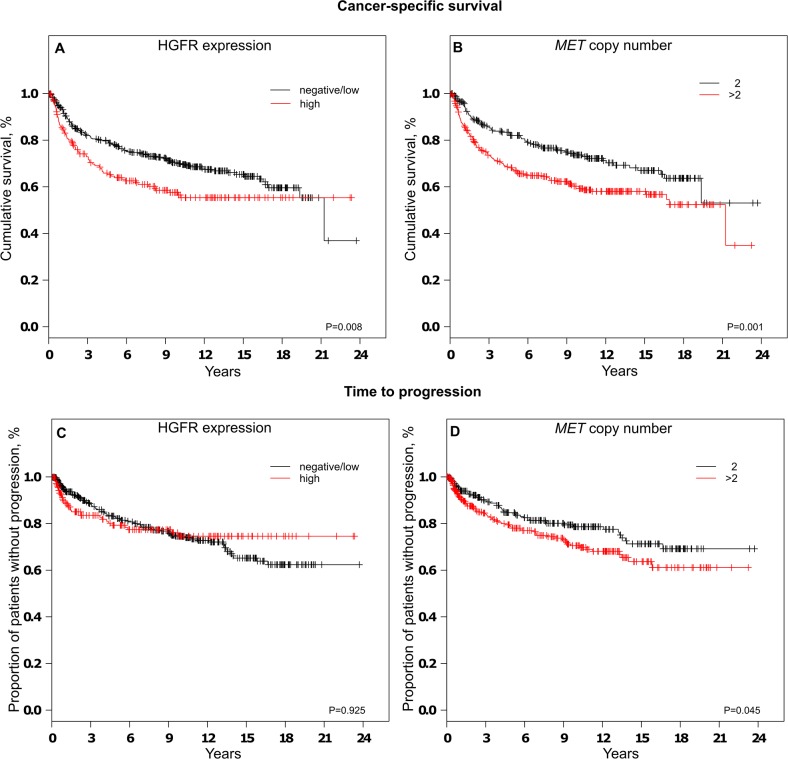
Analysis of cancer-specific survival **A+B** and time to progression **C+D** depending on HGFR expression **A+C** or *MET* copy number status **B+D**

When tumors were grouped according to HGFR expression, univariate survival analysis revealed a decrease in cancer-specific survival (P = 0.008) in patients affected by tumors with high HGFR expression compared to tumors with low/no HGFR expression. In contrast, no consistent relationship was observed between HGFR expression and time to progression (TTP). When tumors were grouped based on *MET* copy numbers per nucleus, cases with more than two *MET* copies exhibited significant shorter cancer-specific survival (P = 0.001) and time to progression (P = 0.046). Kaplan-Meier plots are depicted in Figure 4.

Differences in cancer-specific survival or time to progression did not remain statistically significant after adjustment for established prognostic factors in the multiple regression analysis (Table 2 and Supplementary Table S1).

**Table 2:** Uni- and multivariate analyses of prognostic factors influencing cancer-specific survival (CSS) in clear-cell RCC

	Univariate				Multivariate			
	M0+M1				M0		M1	
	HR(95% CI)	P	HR (95% CI)	P	HR(95% CI)	P	HR (95% CI)	P
Grade of malignancy^1^	**4.7** (3.45-6.39)	<0.001	**2.08** (1.45-2.97)	**<0.001**	**2.58** (1.58-4.22)	**<0.001**	**1.94** (1.14-3.3)	**0.014**
Primary tumor ^2^	**3.9** (2.9-5.24)	<0.001	**2.23** (1.59-3.12)	**<0.001**	**2.79** (1.84-4.22)	**<0.001**	1.3 (0.73-2.34)	0.36
Lymphnode metastasis^3^	**5.24** (3.52-7.78)	<0.001	**1.65** (1.06-2.57)	**0.026**	**2.61** (1.4-4.85)	**0.002**	1.41 (0.74-2.68)	0.29
Distant metastasis^4^	**11.2** (8.23-15.22)	<0.001	**7.0** (4.98-9.84)	**<0.001**	-	-	-	-
ECOG^5^	**0.5** (0.38-0.67)	**<0.001**	**0.68** (0.51-0.91)	**0.01**	0.75 (0.5-1.13)	0.17	0.69 (0.43-1.11)	0.13
Age^6^	1.1 (0.82-1.47)	0.54	1.13 (0.83-1.53)	0.43	1.13 (0.76-1.7)	0.53	1.18 (0.71-1.95)	0.53
Sex^7^	**0.68** (0.5-0.91)	**0.01**	0.81 (0.60-1.11)	0.19	0.67 (0.45-1.0)	0.052	0.91 (0.55-1.5)	0.7
HGFR-Expression^8^	**1.49** (1.11-2.0)	**0.008**	1.02 (0.73-1.43)	0.89	1.05 (0.69-1.61)	0.81	0.84 (0.47-1.5)	0.56
*MET* copy number^9^	**1.62** (1.2-2.18)	**0.001**	1.15 (0.82-1.61)	0.42	0.83 (0.54-1.27)	0.39	1.7 (0.94-3.08)	0.08

1 G3/4 vs G1/G2.

2 pT3/pT4 vs pT1/pT2.

3 pN1/pN2 vs pNx/pN0

4 M1 vs M0.

5 0 vs ≥1

6 > 65 vs ≤ 65

7 Female vs Male

8 high vs negative/low

9 >2 vs 2

HR, hazard ratio; CI, confidence interval. Probability values and hazard ratios considered statistically significant are shown in bold.

## DISCUSSION

Despite comprehensive translational research efforts in renal cell carcinoma and availability of targeted therapy options, at present neither prognostic nor predictive biomarkers are established for routine clinical treatment stratification [28]. Independently to the lack of molecular therapy prediction, targeted therapy has demonstrated modest benefit [29]. However, the rarity of cures and intrinsic or acquired resistance demands novel treatment approaches and identification of predictive and prognostic biomarkers. c-Met/hepatocyte growth factor receptor (HGFR) encoded by the *MET* oncogene is a validated therapeutic target for a number of malignancies and results obtained from clinical trials are encouraging [30].

Our results demonstrate that elevated HGFR protein levels are associated with dedifferentiation and distant metastasis and *MET* copy number gains with dedifferentiation, tumor extent, lymph node, and distant metastasis. Limitations of this study are the retrospective single-institution design and the use of tissue micro array technique, which enables studies on large collectives, but may disregard tumor heterogeneity. However, our findings are in accordance with previous reports showing that elevated HGFR expression correlates with worse cancer-specific survival [25]. Moreover, we demonstrate that *MET* copy number gains correlate with unfavorable patient outcome. In addition, a recent study on brain metastasis of ccRCC indicated MET overexpression as an independent prognostic factor for brain metastasis-specific survival [31].

At present, reliable data regarding diagnostic criteria and predictive cut offs for anti-MET treatment are not available for ccRCC, thus further studies have to define applicable parameters. Recently published final results of the phase 3 trial METEOR comparing cabozantinib (an oral inhibitor of tyrosine kinases including MET, VEGFR, and AXL) versus everolimus in patients with advanced renal cell carcinoma [32] do not provide evidence for a significant relationship between MET expression levels and treatment outcomes. Unfortunately, HGFR expression was only investigated in a subset of RCCs and *MET* copy number status was not determined. Schoffski et al. reported that ALK/ROS/MET inhibitor Crizotinib induced long lasting disease control in metastatic papillary renal cell carcinoma type 1 with *MET* mutation and long term stable disease in a case with *MET* amplification. In contrast, among patients with papRCCs devoid of *MET* amplification or mutation, none achieved a response upon Crizotinib treatment [33].

The MET pathway is associated with breast cancer progression and anti-Met therapies are currently evaluated in breast cancer patients [34, 35, 36]. A phase II study of Foretinib (an oral multi-kinase inhibitor of MET, RON, AXL, TIE-2, and VEGF receptors) in patients with triple-negative breast cancer observed a clinical benefit rate of 46 %. Unfortunately, only 6.7 % of tumors were MET positive by IHC and none of the tumors harbored a *MET* amplification. Hence, the study was unable to confirm MET status as a predictive biomarker [37].

In non-small cell lung cancer the results are inconsistent [38], however HGFR protein levels have been reported to be predictive for anti-MET treatment in clinical studies [39, 40, 41] whereas these results were not confirmed in a phase III trial (METLung) [42]. In contrast, *MET* amplification seems to be predictive based on recently reported results of a Crizotinib phase I study [43].

At present it remains unclear, if *MET* copy number assessment or examination of HGFR protein expression is the approach of choice to identify potential therapy responder. Due to discrepancies in protein levels and copy number status in a subset of tumors, we recommend to perform both tests, *in situ* hybridization and immunohistochemistry, until reliable data is available.

Our results reveal that MET is deregulated in a subset of ccRCC and *MET* copy number gains (>4/nucleus) occur in 7% of therapy-naive ccRCC. Importantly, this proportion increases to 20% in patients with high-grade carcinomas. Naturally, tumor spreading and consecutive need for systemic therapy is more likely in patients with high-grade ccRCC. Furthermore, we identified elevated HGFR expression in 6 and *MET* copy number increase in 5 metastases out of 15 ccRCCs without elevated expression of HGFR or copy number gains of *MET* in the primary tumor. These findings indicate that the MET pathway may be a promising target especially in ccRCC patients with high grade or metastasized disease and the MET status should be re-evaluated in recurrences or metastasis.

*MET* is transcriptionally activated by hypoxia and acts as mediator of antiangiogenic therapy resistance in models of solid tumors [44, 45]. Importantly, also in Sunitinib-resistant RCC, increase in MET expression and activation was observed [46]. Therefore, therapeutically targeting of MET may prevent or overcome antiangiogenic therapy resistance in RCCs. This implies that not only therapy-naive ccRCC with primary MET upregulation are in the focus of therapeutic MET inhibition but also Sunitinib-resistant RCCs. Further studies on RCCs with prior antiangiogenic therapy have to investigate if *MET* amplification is seen in therapy resistant RCCs, resembling a resistance mechanism evident in lung cancers with EGFR activating mutations treated with Gefitinib [47].

Association with clinical and pathological features in primary tumor, *MET* copy number gains in the context of metastatic spread and initial *in-vitro* data indicating that MET-signaling acts as mediator of antiangiogenic therapy [46], suggest a biologic relevance of MET signaling in ccRCCs.

Several compounds including small-molecule TKIs, monoclonal antibodies, and anti-HGF compounds are available for selective inhibition of MET signaling [48, 49] or inhibition of MET in combination with vascular endothelial growth factor receptor (VEGFR) [32, 50] and are already tested in preclinical and clinical trials [51], making anti-MET therapy a near-term feasible treatment option.

In conclusion, our findings highlight inhibition of MET signaling pathway as a promising new target for the treatment of ccRCC patients and prognostic significance of the molecular status of MET. Furthermore, HGFR expression and *MET* copy numbers should not only be assessed in the primary tumor, but also, maybe even more important, in recurrences or metastasis to guide anti-MET therapy in ccRCC patients.

## PATIENTS AND METHODS

### Patients

Tissue samples from 932 patients with primary renal cell carcinomas treated at the Department of Urology at the University of Heidelberg between 1987 and 2005 were collected. The human tissue samples were provided by the Tumour Tissue Bank of the National Centre for Tumour Diseases Heidelberg after approval by the Ethics Committee of the University of Heidelberg. As described previously, clinical follow-up was available for 911 patients, who were prospectively evaluated every 3 months for the first 2 years after treatment, every 6 months for the next 3 years, and yearly thereafter (chest x-ray or thoracic computed tomographic (CT) scan; abdominal sonography or CT scan or magnetic resonance imaging; serum chemistry). No adjuvant treatment of localized disease was administered. Patients with metastasized disease and with a Karnofsky performance index of ≥80 and no medical contraindications received palliative interferon-alpha– and IL-2–based immunotherapy. No targeted therapeutic approaches were performed [52].

### Tissue-Micro-Array

A tissue microarray containing 932 primary tumor and corresponding normal tissue samples of 932 patients was composed. The tumors were graded according to the three-tiered nuclear grading system recommended by the WHO Classification of Tumours 2004 and pathologically staged based on the TNM classification (2009). In total, a set of 19 array blocks was generated, each containing 200 tissue core specimens, representing 50 patients per array. A morphologically representative region was chosen from each of the renal cell carcinomas and two cylindrical core tissue specimens per tumor block measuring 0.6 mm in diameter were punched from these regions and arrayed into the recipient paraffin block. Further details have been described previously [53].

In addition, together with the Tumor Tissue Bank of the National Centre for Tumor Diseases Heidelberg, a second tissue microarray was composed containing tumor tissue of 18 patients with metastasized RCCs. Briefly, representative regions of the primary tumor and the matching metastasis was compiled; three (2x primary tumor, 1x metastasis) cylindrical cores (diameter 1 mm) were punched from the donor blocks and arrayed into the recipient paraffin block using a semiautomatic system (Beecher Instruments, Silver Spring, MD, USA). The origins of metastases were adrenal gland (2x), bone (2x) and lymph node (14x).

### Immunohistochemistry

After heat-induced antigen retrieval using the target retrieval solution ULTRA Cell Conditioning (ULTRA CC1; Ventana Medical Systems, Tucson, AZ, USA; 950-224) tissue microarray slides were stained with an anti-total c-MET (SP44) rabbit monoclonal primary antibody (Ventana Medical Systems; 790-443). Staining was performed using an automated staining system BenchMark ULTRA (Ventana Medical Systems) in accordance with the manufacturer's instructions, the following solutions were used: OptiView DAB IHC Detection Kit (760-700), Hematoxylin I (790-2208), Bluing Reagent (760-2037). The arrays were independently scored by two pathologists (S.M.-G. and W.R.) blinded to tissue annotations and patient outcomes. For the immunohistochemical semiquantitative assessment of HGFR expression, the product of the scores of staining intensity and quantity of immunoreactive tumor cells was calculated based on the following scoring system: the intensity ranged from 0 = negative, 1 = low, 2 = medium to 3 = high; the quantity comprised 0 = no expression, 1 < 10% of positive cells, 2 = positivity in 10% to 50%, 3 = positivity in 51% to 80%, and 4 = positivity in more than 80%. The final immunoreactive score (IRS) score (ranging from 0 to 12) is obtained by multiplication of the intensity score and the quantity score.

### Chromogenic in situ hybridization (CISH)

The commercial available Probe (ZytoDot 2C SPEC MET/CEN 7 Probe, ZytoVision, Bremerhaven, Germany; C-3057-400) a mixture of a Dinitrophenyl-labeled CEN 7 probe specific for the alpha satellite centromeric region of chromosome 7 (D7Z1) and a Digoxigenin-labeled probe specific for the *MET* gene at 7q31 has been used according to the manufacturer's instructions.

### Mutation analysis

Mutation analysis was performed as described in detail previously [54]. Briefly, for library preparation, the multiplex PCR-based Ion Torrent AmpliSeqTM technology (Life Technologies) with the Cancer HotSpot Panel v2 (IonTorrent / Thermo Fisher Scientific, Waltham, USA) was used. Amplicon library preparation was performed with the Ion AmpliSeq Library Kit v2.0 using approximately 10 ng of DNA. Briefly, the DNA was mixed with the primer pool, containing all primers for generating the 207 amplicons and the AmpliSeq HiFi Master Mix and transferred to a PCR cycler (BioRad, Munich, Germany). After the end of the PCR reaction, primer end sequences were partially digested using FuPa reagent, followed by the ligation of barcoded sequencing adapters (Ion Xpress Barcode Adapters, Life Technologies). The final library was purified using AMPure XP magnetic beads (Beckman Coulter, Krefeld, Germany) and quantified using qPCR (Ion Library Quantitation Kit, Thermo Fisher Scientific, Waltham, USA) on a StepOne qPCR machine (Thermo Fisher Scientific, Waltham, USA). The individual libraries were diluted to a final concentration of 100 pM and eight to ten libraries were pooled and processed to library amplification on Ion Spheres using Ion PGMTM Template OT2 200 Kit. Unenriched libraries were quality-controlled using Ion Sphere quality control measurement on a QuBit instrument. After library enrichment (Ion OneTouch ES), the library was processed for sequencing using the Ion Torrent 200 bp sequencing v2 chemistry and the barcoded libraries were loaded onto a chip. Our way of pooling eight samples on a 318 chip resulted in a mean coverage of 3000 fold per amplicon.

### Variant calling and annotation

Data analysis was performed using the Ion Torrent Suite Software (version 4.4). After base calling, the reads were aligned against the human genome (hg19) using the TMAP algorithm within the Torrent Suite. Variant calling was performed with the variant caller plugin within the Torrent Suite Software and the IonReporter package using a corresponding bed-file containing the coordinates of the amplified regions. Only variants with an allele frequency > 5% and minimum coverage > 100 reads were taken into account. Variant annotation was performed using Annovar [55]. Annotations included information about nucleotide and amino acid changes of RefSeq annotated genes, COSMIC and dbSNP entries as well as detection of possible splice site mutations. For data interpretation and validation, the aligned reads were visualized using the IGV browser (Broad Institute) [56].

### Statistical methods

Survival was calculated from the date of nephrectomy to two different events: cancer-specific survival (CSS, event: tumor-related death, survival time was censored for patients who did not experience the investigated event) and time to progression (TTP, event: recurrence, metastasis, deaths before progression were censored). Association between survival times and HGFR expression / *MET* copy number increase was first assessed by log-rank tests and represented using Kaplan-Meier plots. In order to account for the influence of established prognostic factors, hazard ratios (HRs) and 95% confidence intervals (CIs) were adjusted for patient gender and age, tumor extent, lymph node metastasis, distant metastasis, grade of malignancy, and ECOG Performance Status in a multiple Cox proportional hazard regression. Data were analysed using the R software package (https://cran.r-project.org/). For count data, Fisher's exact test (two-sided) was used. Spearman's rank correlation was estimated to quantify the relationship between *MET* copy numbers and HGFR expression. Probability values <0.05 were considered to indicate a statistically significant result.

## SUPPLEMENTARY FIGURE AND TABLE


