# Supplementary material for: MET expression and copy number status in clear-cell renal cell carcinoma: prognostic value and potential predictive marker
Source: Oncotarget. 2016 Nov 24;8(1):1046–57. doi: 10.18632/oncotarget.13540 (PMC5352033; doi:10.18632/oncotarget.13540)
Supplement: Supplementary file 1 [file oncotarget-08-1046-s001.pdf]

MET expression and copy number status in clear-cell renal cell carcinoma: prognostic value and potential predictive marker

SUPPLEMENTARY FIGURE AND TABLE

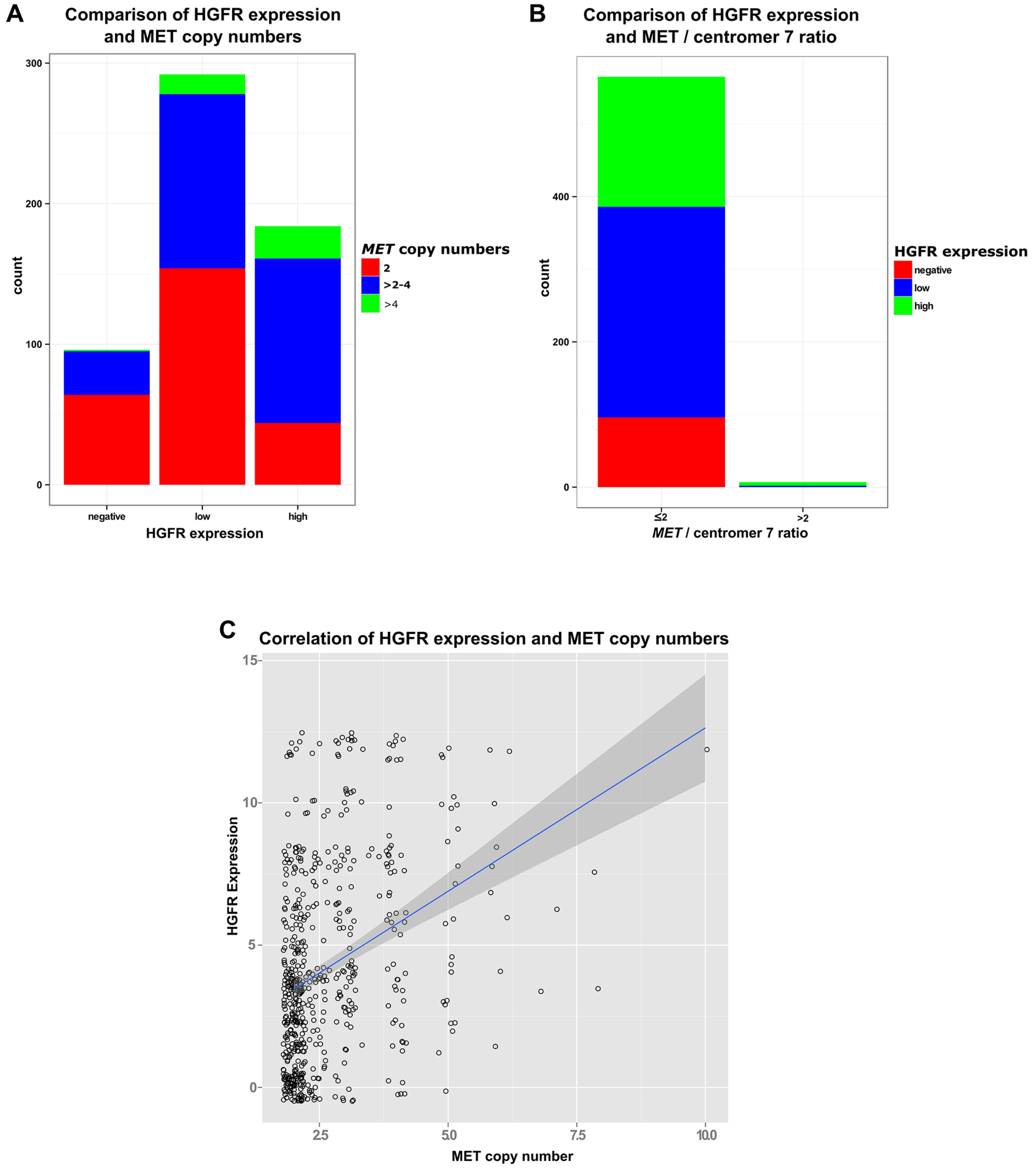

Supplementary Figure S1: A. Comparison of HGFR expression and *MET* copy number. **B.** Comparison of HGFR expression and *MET* copy numbers/centromer 7 ratios. **C.** Scatterplot with regression line (95% CI) depicting relationship of HGFR expression and *MET* copy number.

Supplementary Table S1: Uni- and multivariate analyses of prognostic factors influencing time to progression (TTP) in clear-cell RCC.

	Univariate		Multivariate	
	**HR(95% CI)**	**P**	**HR (95% CI)**	**P**
Grade of malignancy[1]	**2.34** (1.49-3.70)	**<0.001**	**1.40** (0.85-2.28)	0.186
Tumor extent[2]	**3.12** (2.19-4.44)	**<0.001**	**2.54** (1.73-3.72)	**<0.001**
Lymphnode metastasis[3]	**5.24** (2.95-9.30)	**<0.001**	**2.83** (1.53-5.23)	**<0.001**
ECOG[4]	0.94 (0.65-1.37)	0.747	1.00 (0.68-1.47)	0.999
Age[5]	1.19 (0.83-1.70)	0.339	1.03 (0.72-1.48)	0.865
Sex[6]	0.74 (0.52-1.06)	0.102	0.73 (0.51-1.06)	0.099
HGFR Expression[7]	0.98 (0.66-1.45)	0.925	0.82 (0.55-1.24)	0.345
MET copy number[8]	**1.44** (1.01-2.05)	**0.046**	1.17 (0.80-1.72)	0.401

1 G3/G4 vs G1/G2.
2 pT3/pT4 vs pT1/pT2.
3 pN1/pN2 vs pNx/pN0
4 0 vs ≥1
5 > 65 vs ≤ 65
6 Female vs Male
7 high vs negative/low
8 >2 vs 2
HR, hazard ratio; CI, confidence interval. Probability values and hazard ratios considered statistically significant are shown in bold.
